# A Comparative Analysis of the Effect of Carbonaceous Nanoparticles on the Physicochemical Properties of Hybrid Polyethersulfone Ultrafiltration Membranes

**DOI:** 10.3390/membranes12111143

**Published:** 2022-11-15

**Authors:** Lubna Jaber, Ismail W. Almanassra, Sumina Namboorimadathil Backer, Viktor Kochkodan, Abdallah Shanableh, Muataz Ali Atieh

**Affiliations:** 1 Research Institute of Sciences & Engineering (RISE), University of Sharjah, Sharjah P.O. Box 27272, United Arab Emirates; 2 Department of Chemistry, College of Sciences, University of Sharjah, Sharjah P.O. Box 27272, United Arab Emirates; 3 Qatar Environment and Energy Research Institute, Hamad Bin Khalifa University, Qatar Foundation, Doha P.O. Box 5825, Qatar; 4 Department of Civil and Environmental Engineering, College of Engineering, University of Sharjah, Sharjah P.O. Box 27272, United Arab Emirates; 5 Chemical and Water Desalination Engineering Program (CWDE), College of Engineering, University of Sharjah, Sharjah P.O. Box 27272, United Arab Emirates

**Keywords:** ultrafiltration membrane, polyethersulfone, comparison study, membrane fouling, MWCNTs, graphene oxide

## Abstract

Numerous studies have been previously reported on the use of nanoscale carbonaceous fillers, such as multi-walled carbon nanotubes (MWCNTs) and graphene oxide (GO), in polymeric ultrafiltration (UF) membranes; however, no insight has been clearly reported on which material provides the best enhancements in membrane performance. In this study, a comparative analysis was carried out to establish a comprehensible understanding of the physicochemical properties of hybrid polyethersulfone (PES) UF membranes incorporated with MWCNTs and GO nanoparticles at various concentrations. The hybrid membranes were prepared via the non-solvent-induced phase separation process and further characterized by field emission scanning electron microscopy and atomic force microscope (AFM). The AFM images showed homogeneous membrane surfaces with a reduction in the membrane surface roughness from 2.62 nm for bare PES to 2.39 nm for PES/MWCNTs and to 1.68 nm for PES/GO membranes due to improved hydrophilicity of the membranes. Physicochemical properties of the hybrid PES membranes were assessed, and the outcomes showed an enhancement in the porosity, pore size, water contact angle, and water permeability with respect to nanoparticle concentration. GO-incorporated PES membranes exhibited the highest porosity, pore size, and lowest contact angle as compared to PES/MWCNTs, indicating the homogeneous distribution of nanoparticles within the membrane structure. PES/MWCNTs (0.5 wt.%) and PES/GO (1.0 wt.%) hybrid membranes exhibited the highest water flux of 450.0 and 554.8 L m^−2^ h^−1^, respectively, at an applied operating pressure of 1 bar. The filtration and antifouling performance of the PES hybrid membranes were evaluated using 50 mg L^−1^ of humic acid (HA) as a foulant at pH = 7. Compared to the bare PES membrane, the MWCNTs and GO-incorporated PES hybrid membranes exhibited enhanced permeability and HA removal. Moreover, PES/MWCNTs (0.5 wt.%) and PES/GO (1 wt.%) hybrid membranes reported HA rejection of 90.8% and 94.8%, respectively. The abundant oxygen-containing functional groups in GO-incorporated PES membranes resulted in more hydrophilic membranes, leading to enhanced permeability and fouling resistance. The antifouling properties and flux recovery ratio were improved by the addition of both nanoparticles. Given these findings, although both MWCNTs and GO nanoparticles are seen to notably improve the membrane performance, PES membranes with 1 wt.% GO loading provided the highest removal of natural organic matter, such as HA, under the same experimental conditions.

## 1. Introduction

Global water scarcity is increasing annually due to many reasons, most importantly, global population increase, industrialization, climate change, and mismanagement of natural water resources [1]. Efficient desalination and wastewater treatment are crucial for minimizing global water scarcity. Filtration processes based on polymeric membranes are among the leading technologies in this field [2]. Ultrafiltration (UF) is considered a low-pressure-driven membrane technology that can efficiently decontaminate proteins, viruses, macromolecules, suspended particles, and organic substances from water [3]. Despite the separation capabilities of polymeric UF membranes in water treatment, membrane fouling is yet a major challenge in its application that decreases water permeability with time and shortens the membrane life span [4]. Accordingly, different approaches have been investigated for improving the UF polymeric membranes fouling resistance and, at the same time, maintaining higher values of membranes’ water flux [5].

Polyethersulfone (PES) is one of the most widely used polymers to fabricate UF membranes however, the hydrophobic nature of PES membranes limits their fouling resistance leading to decreased water permeability with time [6]. Therefore, porosity and hydrophilicity of PES UF membranes is suggested to be improved by the use of several kinds of fillers [7]. Mixing the PES with certain additives was reported to alter the membrane characteristics such as permeation and fouling properties and mechanical stability [8,9]. In addition, the surface charge of the PES membrane could be rectified toward the removal of certain pollutants [6].

Different materials have been incorporated within PES UF membranes, such as graphene oxide (GO) [10], calcium chloride [11], titania [12], iron oxides [6], hydroxyapatite/boron nitride [13], activated carbon (AC) [14], and multi-walled carbon nanotubes (MWCNTs) [15]. For example, Kallem et al. found that hydroxyapatite-decorated AC nanocomposite improved the hydrophilicity and antifouling properties of bare PES membranes with a maximum flux of 660 L m^−2^ h^−1^ using 4 wt.% of nanoparticles [14]. Recently, it was found that iron oxide-functionalized halloysite nanoclay can improve the properties of PES UF membranes with a maximum flux of 294.4 L m^−2^ h^−1^ using 0.1 wt.% of the prepared composite [16]. Among the aforementioned materials, carbon-based materials such as GO and MWCNTs were of great interest in altering the physiochemical properties of PES UF membranes. GO and MWCNTs are chemically and mechanically stable, accessible and have been widely reported to improve the antifouling properties and water permeability of PES membranes. For instance, Algamdi et al. reported an increase in the water flux with increasing GO loading reaching a maximum flux of 340 L m^−2^ h^−1^ using 5 wt.% of GO nanoparticles [10]. Moreover, their results demonstrated a gradual decrease in membrane contact angle and better humic acid (HA) removal by increasing the GO loading [10]. In another report, Najjar et al. prepared PES UF membranes using Arabic gum (1 wt.%) as a pore-forming agent and different loadings of oxidized MWCNTs. The filtration outcomes illustrated an increase in water permeability up to 500 L m^−2^ h^−1^ using 3 wt.% of MWCNTs. Additionally, promising results were obtained in total fouling resistance and mechanical strength for the modified membranes [15]. Many other reports concluded that the use of carbon nanoadditives improved the permeability and antifouling characteristics of polymeric UF membranes [17,18].

Despite the extensive research progress made to improve the physiochemical properties of PES UF membranes by MWCNTs and GO, it is yet incredibly difficult to compare the effect of embedding nanoadditives within PES matrices to evaluate the membrane’s performance. For example, some studies reported the suitable distribution of GO nanosheets and MWCNTs nanoparticles in polymeric membranes [10,15], while other studies reported the weak distribution and agglomeration of GO nanoparticles within the membrane structure [19]. Therefore, it is necessary to provide the readers and scientific community with a comparative study on the effect of GO and MWCNTs nanoadditives on the physiochemical and antifouling properties of PES UF membranes using the same fabrication and testing conditions. There are very limited studies in the literature that compares GO and MWCNTs as nanoadditives in polymeric membrane applications [20,21]. Seetharaman et al. found that GO is a better filler for polysulfone polymer than MWCNTs in water electrolysis applications [20]. Another study by Zhang et al. reported that oxidized MWCNTs provide lower contact angle and higher water flux for polyvinylidene fluoride ultrafiltration membranes compared to GO-modified UF membranes; however, this study only focused on studying the synergetic effect of GO/MWCNTs mixtures at different mass ratios, and hence, no comparative conclusions between GO and MWCNTs were laid out at the end [21].

The main objectives of the current study are: (i) synthesize GO nanosheets by the Hummer’s method and investigate the morphology and surface properties of raw GO and MWCNTs, (ii) inspect the effect of mass loading of GO and MWCNTs on the water flux and antifouling properties of PES UF membranes, (iii) explore the effect of GO and MWCNTs on the morphology and structure of PES UF membranes, (iv) examine the influence of GO and MWCNTs on the water content, porosity, and contact angle of PES UF membranes, (v) study the HA removal by bare PES, GO, and MWCNTs-modified UF membranes, and, finally, (vi) based on the obtained data, comparison conclusions on the effect of incorporation of MWCNTs and GO nanoadditives on the performance of - PES UF membranes were drawn.

## 2. Materials and Methods

### 2.1. Materials

Graphite nanopowder (particle size: 300 nm) was purchased from Changsha Easchem Co., Limited, Changsha, China. Multi-walled carbon nanotubes (MWCNTs, length 10–30 μm and outer diameter 20–40 nm) were obtained from Chengdu Organic Chemicals Co. Ltd., Chengdu, China). Polyethersulfone pellets (PES; M_W_: 59 kDa) were purchased from GoodFellow Co., Cambridge, England, U.K. Polyvinylpyrrolidone (PVP; M_W_: 360 kDa) was acquired from Sigma Aldrich, Beijing, China. Humic acid (HA, M_W_: 227.17 Da) was obtained from Sigma Aldrich, Steinheim, Germany. N,N-dimethylacetamide (DMA), sulfuric acid (H_2_SO_4_), hydrochloric acid (HCl, 37%), sodium nitrate (NaNO_3_), hydrogen peroxide (H_2_O_2_, 35%) and potassium permanganate (KMnO_4_) were obtained from Merck, Darmstadt, Germany. The non-solvent used in the coagulation bath was Milli-Q deionized water (DW, 0.055 µS cm^−1^). All chemicals were of analytical reagent grade and were used without further treatment.

### 2.2. Synthesis of Graphene Oxide

In brief, 2 g of graphite nanopowder and 1 g of NaNO_3_ were added into a 500 mL volumetric flask containing 90 mL ice-cold concentrated H_2_SO_4_. The mixture was stirred continuously for 10 min to ensure uniform dispersion. Next, 12 g of KMnO_4_ was gradually added to the mixture while maintaining a temperature below 5 °C using an ice bath. The mixture was then stirred for approximately 2 h while maintaining a temperature of 45 °C using an oil bath until the mixture turned pasty brown. A total of 100 mL DW was then added in a dropwise fashion to the mixture while maintaining a temperature below 20 °C. Next, stirring was continued for 1 h at 85 °C. Later, to arrest the reaction, 120 mL chilled DW was added to the mixture, followed by the slow addition of 15 mL 30% H_2_O_2_, which, as a result, caused a color change to bright yellow. The sample was then centrifuged at 5000 rpm for 30 min at room temperature and washed with 5% HCl to remove existing impurities. The supernatant was then discarded, and the process was repeated several times using DW to neutralize the content and regenerate the pellet. After the neutral pH was achieved, the GO pellet was dried overnight in an oven at 85 °C and then ground into powder form and kept for further analysis.

### 2.3. MWCNTs and GO Characterization

The nano-fillers (MWCNTs and GO) were characterized through the measurements of specific surface area and pore size, surface functional groups, crystallographic structure, morphology, and elemental analysis. The surface area and pore size distribution measurements were obtained from the nitrogen adsorption/desorption technique using the NOVATECH LX2 BET analyzer from Anton Paar GmbH, Graz, Austria. The functional groups on MWCNTs and GO nanomaterials were identified using a Fourier Transform Infrared spectroscopy (FTIR) using the JASCO FTIR-6300 instrument, Jasco Inc., Tokyo, Japan. The samples were prepared by the KBr pellet method, and the spectral range was investigated between 4000 and 400 cm^−1^. A powder X-ray diffractometer (XRD, D8 Advance, Bruker, Bremen, Germany) was utilized to evaluate the crystallographic structures of MWCNTs and GO nanomaterials. Furthermore, morphological, and elemental analysis was conducted via a Field emission scanning electron microscopy (FESEM, type: Apreo, Thermo Fisher Scientific, Waltham, MA, USA) fitted with Energy Dispersive X-ray Spectroscopy (EDS, Bruker Xflash 6/60, Berlin, Germany). The number of graphitic layers in the prepared GO was studied by using Raman spectroscopy within a spectral range between 200 and 3000 cm^−1^ (RENISHAW Raman Microscope, Wotton-under-Edge, England, UK) and an atomic force microscope (AFM, Nanosurf 3000, Liestal, Switzerland) equipped with Gwiyddion software was utilized to process the AFM height images and the thickness profiles.

### 2.4. Membrane Fabrication

Membrane casting solutions were prepared by mixing PES (16 wt.%) and PVP (2 wt.%) in DMA solvent (84 wt.%). Varying quantities of the nanoadditives (elaborated in Table 1) were added to the doped solutions to cast the PES membranes. The casting solutions were first stirred for 1 h using a digital hotplate stirrer (Daihan scientific, Wonju, South Korea), followed by probe sonication (Hielscher Ultrasonics, UP400St, Teltow, Germany) at a frequency of 24 kHz for a period of 1 h to allow the homogeneous dispersion of the additive materials throughout the solution. The solutions were then degassed for 1 h to eliminate any air bubbles. The membrane casting was carried out on a glass plate at room temperature following the standard non-solvent-induced phase separation technique using a knife casting system (Automatic thick film coater, MSK-AFA-II, MTI corporation, Qingdao, China). All membranes were cast at a speed of 2.7 m/min (45 mm/s) and a knife gap height of 250 µm. The glass plate was then gently immersed into a coagulation bath containing DW, allowing the exchange of DMA/DW solvents. This technique allows the precipitation of the polymeric membrane of the glass. Lastly, the fabricated membranes were washed several times and stored in DW until further testing.

### 2.5. Membrane Characterization

Surface and cross-section morphologies of the prepared membranes were evaluated using FESEM. The cross-section membrane samples were fractured in liquid nitrogen, while other samples were coated with a gold layer using a sputtering coating machine prior to the analysis. Vacuum conditions were set throughout the analysis to demonstrate high-resolution images. In addition to FESEM imaging, elemental identification of the membrane samples was demonstrated using EDS analysis. AFM was used to study the topography of the membranes. Moreover, Gwiyddion software was utilized to process the AFM images and assess the roughness of the membrane surface.

The porosity (ε,%) and water uptake (φ, %) of the membranes was assessed using gravimetric methods [22]. Triplicate square samples of each membrane were cut to a set measured size (2 × 2 cm) and weighed using an analytical balance. The pieces of the membrane were soaked in DW at room temperature for 24 h. Excess water droplets were carefully wiped off before weighing the membrane to obtain the wet mass ($w_{w}$). The membranes were then dried in the oven at 50 °C for 24 h before obtaining the dry mass ($w_{d}$). The average porosity and the water uptake were calculated using Equations (1) and (2) [22]:

(1) $$\varepsilon \;\left(\%\right)=\frac{w_{w}-w_{d}}{A\times l\times \rho }\times 100\;$$

(2) $$\varphi \;\left(\%\right)=\frac{w_{w}-w_{d}}{w_{d}}\;$$

where $w_{w}\;$ and $w_{d}\;$are the wet and dry masses of the membranes, respectively, ρ is the density of DW at 25 °C, *A* is the area of the membrane (cm^2^), and *l* is the thickness of the membranes (cm). Using the Guerout–Elford equation (Equation (3)) and the values of the membrane porosity and flux, the mean pore sizes of the membranes can be calculated as follows [23]:

(3) $$r_{m}=\sqrt{\left(\frac{8\times µ\times l\times Q\times \left(2.9-1.75\times \varepsilon \right)}{\varepsilon \times A\times \Delta P}\right)\;}\;$$

where ε is the porosity (%), Q represents the volume of DW (m^3^/s), µ is the viscosity of DW at 25 °C taken as 8.9 × 10^−4^ Pa. s, and P is the operating pressure (typically 1 bar). 

A Ramé-hart contact angle goniometer (USA) was used to measure the water contact angle (WCA) between the water droplet and the surface of the membrane with a drop size of 2.5 µL. Contact angle measurements were taken at 3 different locations on the membrane surface for every membrane sample.

The degree of interaction between the water droplets and the membrane surface can be measured through the surface free energy ($-\Delta G_{LS}$) from the static contact angle using the Young–Dupre equation (Equation (4)) stated below [24]:

(4) $$-\Delta G_{LS}=\left(1+COS\theta \right)\times \gamma_{L}\;$$

where $\gamma_{L}\;$is the surface tension of water taken as 72.8 mJ m^−2^.

### 2.6. Ultrafiltration and Water Flux Assessment

Flux measurements and membrane performance evaluation were carried out using a high-pressure 316 stainless steel stirred dead-end filtration cell (HP4750, Sterlitech, Auburn, AL, USA) with an active membrane surface area of 14.6 cm^2^. The cell was connected to an air cylinder comprising a pressure regulator to control the applied operating pressure on the cell to the feed. Membrane water flux ($J_{w}$, L m^−2^ h^−1^) was calculated using the collected permeate volume at a particular time interval using Equation (5).

(5) $$J_{w}=\frac{V}{A\times t}\;$$

where V is the permeate volume (L), A is the area of the membrane (m^2^), and t is the time required to collect the water (min).

The performance of the PES hybrid membranes was evaluated by the dead-end continuous filtration cell at room temperature. Briefly, 250 mL DW was filled in the cell and filtered at a stirring rate of 200 rpm by applying an operating pressure of 1–5 bar. The HA rejection and fouling experiments were conducted through the filtration of 250 mL of 50 mg L^−1^ HA solution at pH = 7. HA experiments were comprised of 20 min DW filtration, followed by additional 20 min HA solution filtration. The membrane was then thoroughly cleaned using DW for approximately 6 min at a stirring rate of 500 rpm. After the cleaning procedure, DW was filtered again through the washed membranes. Three consecutive filtration cycles were carried out for selected PES membranes. HA flux and normalized flux of the PES hybrid UF membranes were calculated using Equations (5) and (6), respectively.

(6) $$Normalized\;flux=\frac{J_{w2}}{J_{w1}}$$

where $J_{w2}$ is the water flux of the membranes after the cleaning procedure and $J_{w1}\;$is the initial water flux of the membrane.

The fouling resistance properties of the membranes during HA filtration can be better interpreted through quantitative variables such as the flux recovery ratio (FRR, %) and irreversible $\left(R_{ir},\%\right)\;$fouling rates determined using Equations (7) and (8), respectively.

(7) $$FRR\left(\%\right)=\frac{J_{w2}}{J_{w1}}\times 100\;$$

(8) $$R_{ir}\left(\%\right)=\left(\frac{J_{w1}-J_{w2}}{J_{w1}\;}\right)\times 100\;$$

For HA rejection tests, filtrate fluxes were recorded for a period of 20 min, followed by collecting 10 mL of the permeate. The optical densities of the feed and collected HA permeate samples were measured using a UV-Vis spectrophotometer (Shimadzu, Nakagyo-ku, Kyoto, Japan) calibrated at an absorption peak of 254 nm. Equation (9) was used to calculate the HA rejection (R, %).

(9) $$R\left(\%\right)=\left(1-\frac{C_{p}}{C_{f}}\right)\times 100\%$$

where $C_{p}\;$and $C_{f}\;$are the concentrations of HA in the permeate and in the feed solution, respectively.

## 3. Results and Discussion

### 3.1. Characterization of MWCNTs and GO

**Figure 1:**
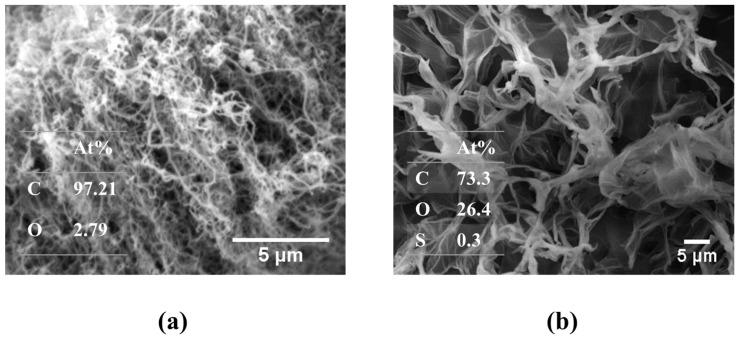
FESEM micrographs of (**a**) MWCNTs and (**b**) GO nanosheets, including their corresponding EDS spectra as insets.

The FESEM micrographs of MWCNTs and GO are presented in Figure 1a,b, respectively. The FESEM results demonstrated that the MWCNTs have a well-defined tubular structure. Moreover, the MWCNTs were found to be randomly oriented and agglomerated in some areas, such as cotton clumps. The GO nanosheets presented an exfoliated sheet-like morphology, indicating successful oxidation of graphite using Hummer’s method. Moreover, the EDS spectra of GO (an inset of Figure 1b) show abundant oxygen content (atomic percentage: 26.4%), while MWCNT demonstrated 2.79 at.% of bulk oxygen content, suggesting the GO particles might demonstrate higher oxygen functional groups than the MWCNTs.

**Figure 2:**
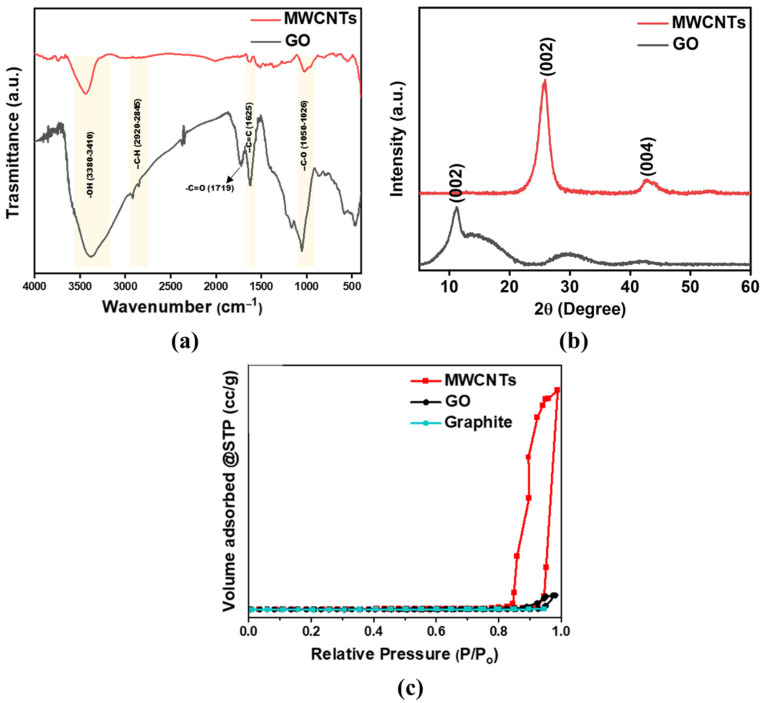
(**a**) FTIR spectra of MWCNTs and GO, (**b**) XRD spectra of MWCNTs and GO, and (**c**) N_2_ adsorption-desorption isotherm of MWCNTs, GO, and graphite.

The FTIR spectra of MWCNTs and GO are depicted in Figure 2a. Both MWCNTs and GO nanoparticles showed a broad peak at around 3410–3380 cm^−1^ of −OH stretching vibrations. The shoulder peaks observed at 2920–2845 cm^−1^ corresponds to −CH stretching vibrations. The presence of aromatic −C=C stretching vibration at 1625 cm^−1^ signifies the graphitic structure of MWCNTs and GO. A sharp peak in the range of 1050–1026 cm^−1^ corresponds to the C-OH vibration of the carboxylic group in both samples. Compared to MWCNTs, GO is found to have a sharp peak at 1719 cm^−1^ related to the carbonyl group vibration. Moreover, the intensities of GO peaks are found to be higher, validating the presence of more oxygen-containing functional groups [25]. The FTIR outcomes confirm the EDS analysis that GO particles illustrated higher amounts of oxygen-containing functional groups.

The powder X-ray diffraction spectra (Figure 2b) of MWCNTs demonstrated two peaks at around 25.9° and 42°, which corresponds to (002) and (100) planes of graphitic and carbon nanotube structure, respectively. The XRD spectra of GO show a strong diffraction peak at 11.2°, which corresponds to the (002) plane of graphitic carbon [25].

The N_2_ adsorption-desorption isotherm of MWCNTs and GO (Figure 2c) can be categorized as a type IV isotherm with an H1 hysterias loop. The appearance of the H1 loop at P/P_0_ of 0.8 to 1 indicates the presence of mesoporous along with macroporous structure [26]. The specific surface area of MWCNTs, GO, and graphite is found to be 149.28, 67.14, and 12.9 m^2^ g^−1^, respectively. The increased surface area of GO compared to graphite indicates the successful oxidation of graphite layers via Hummer’s method. The oxidation can lead to the exfoliation of graphitic layers, which imparts the surface area of GO [27,28]. In addition, the exfoliation of GO layers was clearly evident in the SEM image of GO.

**Figure 3:**
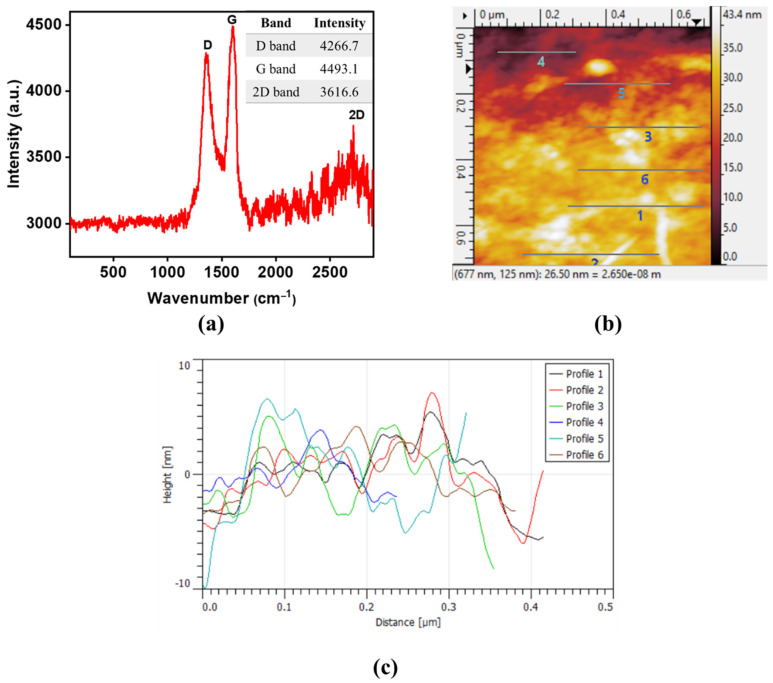
(**a**) Raman spectra of GO, including their corresponding band intensities as an inset, (**b**) AFM height image of GO sheets, and (**c**) AFM section profiles of GO sheets analyzed at different areas.

The number of graphitic layers on the synthesized GO was investigated using Raman spectroscopy (I_2D_/I_G_ ratio) and AFM analysis, as per the previous literature [29]. From the Raman spectra of GO (see Figure 3a), the intensity ratio of I_2D_/IG is found to be 0.85, which indicates the formation of a few layers (4–5 layers) of GO. These outcomes are in agreement with previously reported studies conducted by Nguyen et al. [30] and Hwangbo et al. [31]. Additionally, the number of layers was measured using AFM cross-section analysis. The AFM thickness measurement provides an idea about the number of layers presented in the GO [29]. Figure 3b displays the height image of the prepared GO, showing sheeted morphology with serval micrometer length as well as some aggregation. This aggregation could be formed during the sample preparation. In order to analyze the number of layers, section profiles were performed using the Gwydion software. Figure 3c shows the thickness of GO sheets in a number of different areas. The results showed that the thickness is in the range of 2–9 nm, which confirmed the multilayer formation (less than 10 layers) in the prepared GO.

### 3.2. Membrane Characterization

**Figure 4:**
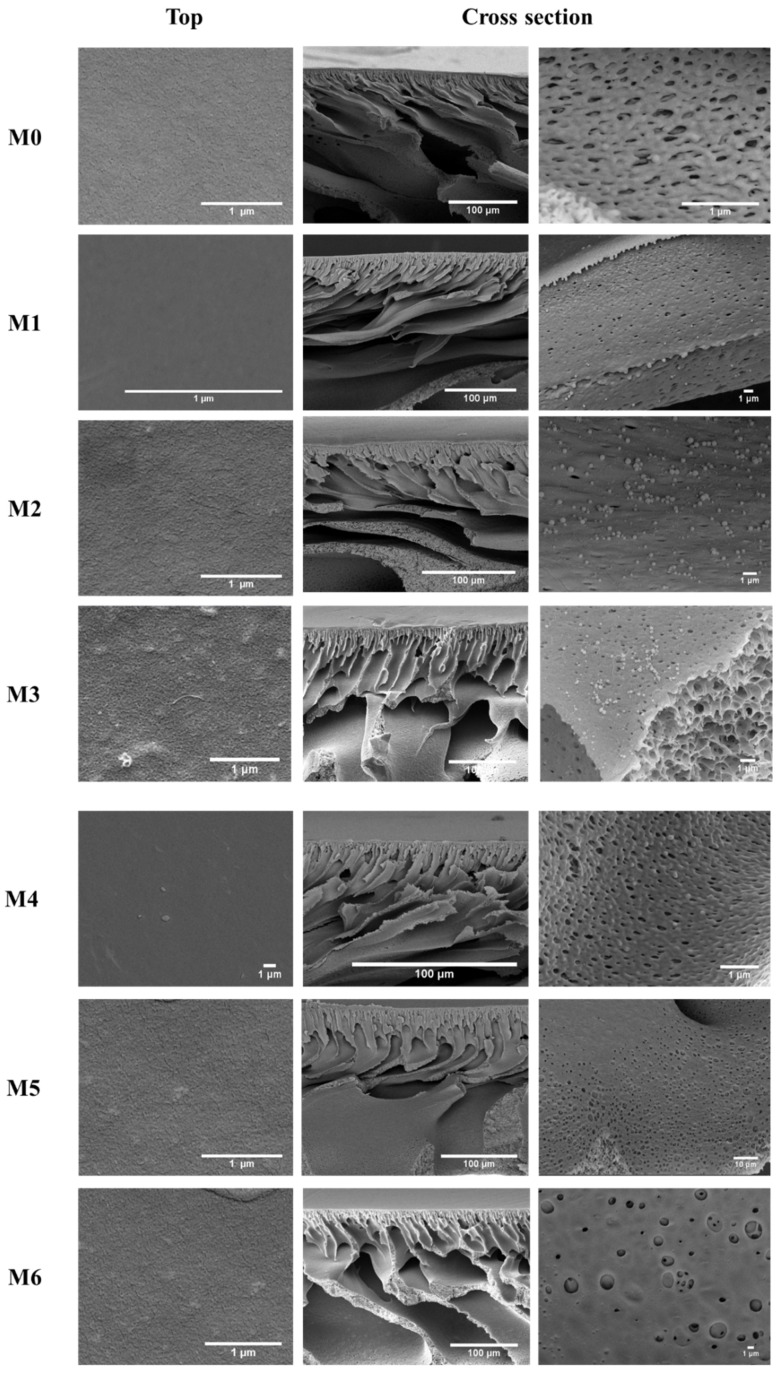
FESEM images of the bare PES, PES/MWCNTs, and PES/GO membranes’ top surfaces and cross-section view.

The morphology changes of the top surfaces and membrane cross-section view of the bare and modified PES membranes were investigated by FESEM. Figure 4 displays the FESEM images of the pristine PES membrane versus the PES membranes incorporated with various loadings of MWCNTs and GO nanoadditives into the polymeric matrix. All fabricated membranes demonstrated an asymmetric morphology with a dense top membrane surface and a larger, loose, and porous bottom layer. Moreover, the membranes also possessed relatively small pores with no visible defects on their surfaces. The bare PES membrane exhibited a very smooth top surface with a sponge-like cross-section, suggesting thermodynamic stability of the primary phase during the phase inversion process. The incorporation of MWCNTs and GO material into the polymer matrix caused the formation of a finger-like cross-section, which can be attributed to the decreased thermodynamic stability as a result of instantaneous demixing between the solvent and non-solvent during membrane preparation [32]. The top surfaces of M1-M6 membranes were found to be regular and smooth, suggesting the homogeneous distribution of MWCNTs and GO nanoparticles within the structure of the PES membranes. According to the magnified cross-section images, MWCNTs were seen to be deposited within the inner walls and pores of the membrane channels, whereas GO nanosheets were embedded within the polymeric matrix during the phase inversion process. Due to the thermodynamic instability of MWCNTs and GO nanoparticles and their migration along the polymer chain to the upper surface of the membranes during the casing process, an increase in pore size and more gaps were noticed in the body of the modified membranes. Consequently, the particles remained homogeneous and well distributed on the inner walls and within the structure of the membranes. Despite the minor changes in the surface and cross-sectional morphology after the addition of MWCNTs and GO nanoparticles, it is expected that the incorporation of these carbonaceous fillers could increase the membrane hydrophilicity and result in improved water passage properties that will be investigated in the following sections.

**Figure 5:**
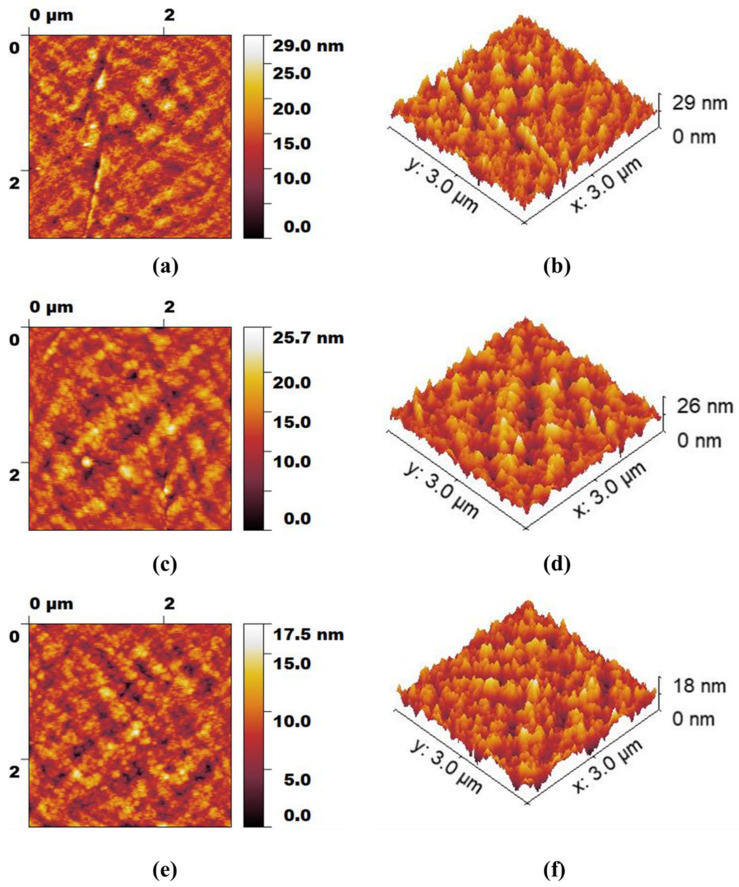
AFM height profiles and 3-dimensional images of M0 (**a**,**b**), M2 (**c**,**d**), and M5 (**e**,**f**) membranes.

The AFM height profiles and 3-dimensional images of M0, M2, and M5 membranes are depicted in Figure 5a–f. All the membranes (3 × 3 µm analysis area) were found to have comparatively smooth homogeneous surfaces. The average surface roughness of M0, M2, and M5 membranes are determined to be 2.62 nm, 2.39 nm, and 1.68 nm, respectively. The lower value of surface roughness for M5 indicates the strong interaction between the PES and GO nanosheets, which in turn provides a much smoother membrane surface as compared to the bare PES and PES/MWCNTs membranes. This can be explained by the presence of a larger number of oxygen-containing functional groups in the GO, which could interact with sulfonyl groups of the PES via hydrogen bonding. In addition, it might be assumed that suitable dispersibility of GO within the PES matrix at low GO loadings might also account for the low surface roughness of the PES/GO membrane. It has been reported that high loadings of carbon material in polymeric membranes could increase the surface roughness owing to the strong electrostatic attraction between the nanoadditives and thereby result in their agglomeration [33,34].

**Figure 6:**
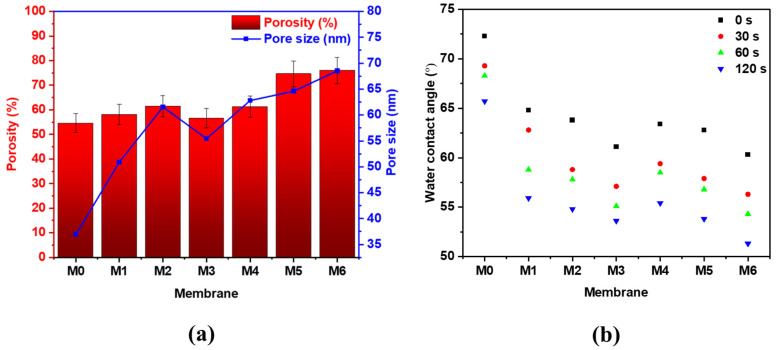
(**a**) Porosity and pore size, and (**b**) WCA at different time intervals of bare PES, PES/MWCNTs, and PES/GO membranes at different loadings of the additives.

The incorporation of MWCNTs and GO to the PES matrix showed a significant enhancement in the physicochemical properties of the PES hybrid membranes, as displayed in Figure 6 and Table 2. The porosity and mean pore size of the PES membranes was observed to increase with the addition of MWCNTs up to 0.5 wt.% (from 54.6% to 61.5% for porosity and from 36.9 nm to 61.6 nm for pore size), after which was seen to decrease slightly. Similar trends were reported by Najjar et al. [15] and Zhu and Wang [35] where a decrease in the porosity and pore size of the membrane was observed with increasing concentrations of MWCNTs. It was reported that at higher loadings, MWCNTs have the ability to aggregate, causing possible blockage of the membrane pores, therefore resulting in a slight decrease in the porosity and pore size. Similarly, the membrane porosity and pore size was found to significantly increase with GO loading. M6 membrane with 1 wt.% GO exhibited the highest porosity (76%) and pore size (68.5 nm), suggesting that GO has a more pronounced effect on the pore formation as compared to MWCNTs. The largest porosity and pore size obtained in M6 membrane might be attributed to the high GO loading and increased hydrophilicity of the dope solution, which greatly affects the substitution rate of DMA solvent with DW during the membrane casting.

Typically, WCA measurements provide an estimate of the wettability and hydrophilicity of the membrane surface. Smaller contact angles indicate improved wettability and, thus, a more hydrophilic membrane surface and vice versa. According to the results displayed in Table 2, a reduction in the contact angle was noticed in GO and MWCNTs incorporated membranes compared to the bare PES membrane, implying an enhanced interfacial free energy and improved hydrophilicity of the hybrid membranes. Moreover, higher loadings of the nanoadditives led to a higher decrease in the WCA. For example, the WCA of membranes with MWCNTs decreased from 64.8° (0.1 wt.%) to 61.1° (1 wt.%), while GO addition led to a decrease from 63.4° (0.1 wt.%) to 60.3° (1 wt.%). Similar outcomes were obtained by Mehrparvar et al. [36], who reported that a higher hydrophilic membrane structure is more likely to increment water uptake and result in higher diffusion of water molecules through the membrane structure. Figure 6b shows the WCA of the bare PES and PES/hybrid membranes studied over a period of 120 s. It was noticed that the WCA for all membranes decreased with time, mainly due to the physicochemical properties of the membranes as well as the presence of oxygen-containing functional groups, which play a great role in the water droplet behavior. PES/MWCNTs and PES/GO membranes showed a higher decrease in the WCA as compared to the bare PES membrane, mainly a result of increasing porosity and pore size of the membranes with increment addition of MWCNTs and GO, as well as the chemical attraction between the water droplet and the surface of the membrane. This facilitates the passage of the water droplet into the membrane’s interior, causing a drop in the WCA with time [14].

As clarified in Table 2, the PES/GO membranes exhibited higher water uptake than MWCNTs incorporated membranes. The higher water uptake for PES/GO membranes could be due to the existence of hydrophilic moieties such as −OH, −COOH, and −O− functional groups of GO. These groups commonly provide additional water storage spaces and enhance the membrane strength to attract water molecules. Furthermore, the polarities of PES and PVP enable them to interact strongly with GO through the hydrophilic moieties during the phase inversion process. Similar trends were reported in a study performed by Algamdi et al. [10].

### 3.3. Membrane Performance

#### 3.3.1. Membrane Fluxes during Filtration of Pure Water and HA Solutions

**Figure 7:**
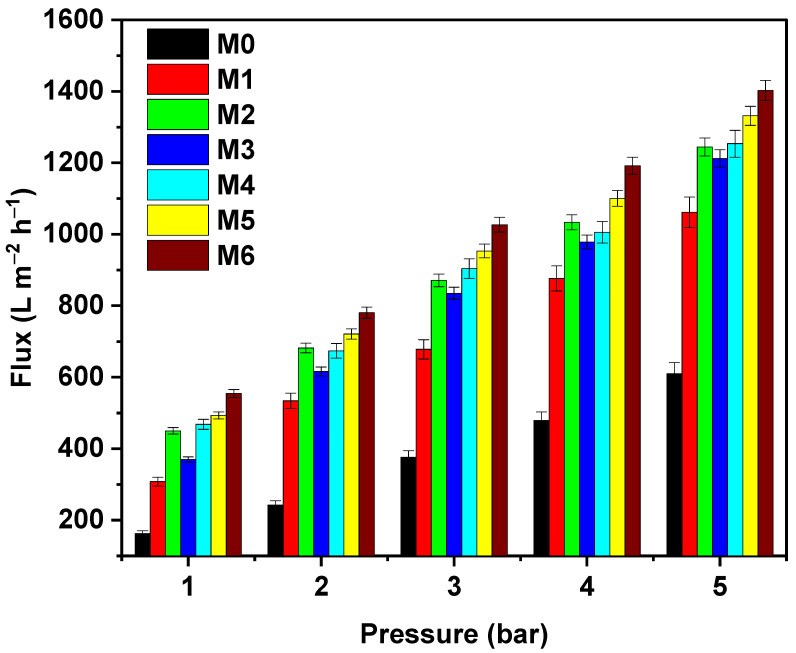
Influence of applied operating pressure on the water flux for bare PES, PES/MWCNTs, and PES/GO membranes.

Figure 7 shows the water fluxes of bare and modified PES membranes at varying operating pressures ranging from 1 to 5 bar. It is seen that the MWCNTs and GO-modified membranes were more permeable than the bare PES membranes. The bare membrane (M0) with the smallest porosity and water uptake and largest contact angle exhibited the lowest water flux compared to MWCNTs and GO-incorporated PES membranes. In the case of MWCNTs-incorporated PES membranes, it was noticed that the water flux was seen to increase from 0.1 to 0.5 wt.% at all operating pressures. When the concentration of MWCNTs in the polymer matrix was 0.5 wt.%, the phase separation was enhanced due to the presence of hydrophilic MWCNTs nanoparticles that caused the formation of larger pores and higher water flux. However, when the loading of MWCNTs was increased beyond 0.5 wt.% (to 1 wt.%), the viscosity of the dope solution increased, which, in turn, resulted in a delayed phase separation process. This behavior led to the formation of smaller pore size and lower water flux. Similar trends were reported by Celik et al. [37] and Han et al. [38]. On the other hand, the GO blend PES membranes possessed a consistent increase in the water flux with GO loadings as well as operating pressures. This can be attributed to the increased porosity, pore size, and hydrophilicity of the PES membranes upon the addition of GO nanosheets containing excessive amounts of oxygen functionalities. A pressure of 1 bar was seen to be sufficient enough to provide a UF membrane flow and was employed in further experiments.

#### 3.3.2. HA Filtration

**Figure 8:**
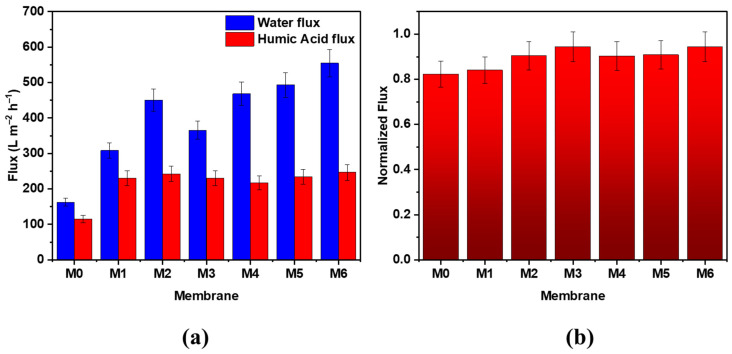
(**a**) Permeate fluxes during filtration of pure water and HA solutions with bare PES, PES/MWCNTs, and PES/GO hybrid membranes, and (**b**) corresponding normalized flux for different PES hybrid membranes.

Figure 8a displays a bar graph showing the water (J_w_) and HA (J_HA_) fluxes of the hybrid membranes comprising different loadings of MWCNTs and GO in the PES matrix compared to the bare membrane. The figure clearly shows a gradual increase in the water flux for the modified membranes as compared to the bare PES membrane. The water flux was seen to increase from M0 to M2 samples upon the addition of 0.1 and 0.5 wt.% MWCNTs, followed by a decrease in the water flux from 450.0 L m^−2^ h^−1^ to 365.8 L m^−2^ h^−1^ when MWCNTs loading is increased from 0.5 wt.% to 1 wt.%. This could be accounted for by the slight decrease in the porosity and pore size of the M3 membrane at high MWCNTs loading. Similar trends were reported by Rahimpour et al. [39], who concluded that by raising the concentration of MWCNTs, the viscosity of the casting solution will increase, and the membrane porous structure becomes denser, which leads to a decrease in the flux. Furthermore, higher MWCNTs loadings will affect the rate of the solvent/non-solvent exchange during the phase inversion process and thus slows down the precipitation of the membrane in the coagulation bath. Consequently, a less porous membrane is formed during the membrane casting. For the GO-modified membranes, the water flux was found to increase from M4 to M6 samples. The M6 membrane exhibited the highest initial water value of 554.8 L m^−2^ h^−1^, which was 3.45-fold higher than the bare PES membrane’s initial water flux. These enhancements can be related to higher hydrophilicity, pore size, and porosity of the M6 sample compared to the bare PES membrane (see Figure 6 and Table 2, respectively). The permeate fluxes during HA filtration with PES/GO and PES/MWCNTs membranes were also notably higher compared to the bare PES membrane. The fluxes of PES hybrid membranes robustly increase with increasing MWCNTs and GO loading. The highest J_HA_ value of 246.6 L m^−2^ h^−1^ was obtained for the M6 membrane. For all prepared membranes, the J_HA_ values were found to be lower than the J_w_ values. This could be a result of the concentration polarization close to the surface of the membrane and membrane fouling. Figure 8b displays the normalized flux for different PES hybrid UF membranes before and after HA filtration. The figure clearly shows an enhancement in the normalized flux after HA filtration for MWCNTs and GO hybrid membranes. An increase in the normalized flux was also noticed with the incremental addition of MWCNTs and GO additives to the PES matrix.

**Figure 9:**
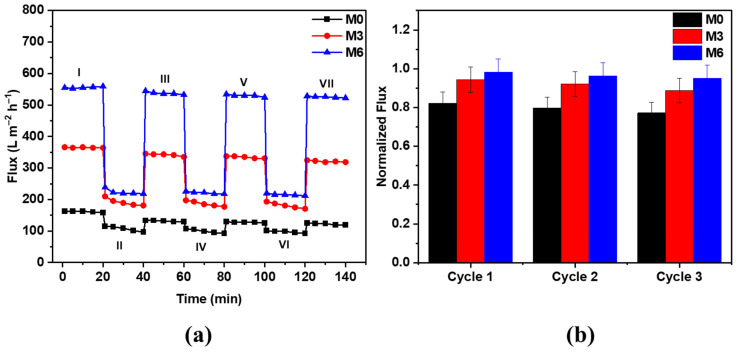
(**a**) Flux modulation of bare PES (M0), PES/MWCNTs (M3), and PES/GO (M6) hybrid membranes during water (phases I, III, V, and VII) and HA filtration (phases II, IV, and VI) where each phase was run for 20 min, and (**b**) corresponding normalized fluxes for different PES hybrid membranes.

Membranes M0, M3, and M6 were selected for further antifouling assessment using HA as the feed solution at an operating pressure of 1 bar. Moreover, the recyclability of the membranes was evaluated by conducting three water and HA filtration cycles. The filtration cycle was comprised of four water filtration phases (I, III, V, and VII) and three HA filtration phases (II, IV, and VI), as indicated in Figure 9a. Each phase was run for a period of 20 min. After each HA filtration phase, the membrane was thoroughly washed with DW for 6 min at a stirring rate of 500 rpm prior to further water filtration. Regardless of the investigated membrane type, a modest reduction in permeate flux persisted even after the membrane was washed, suggesting membrane fouling. The M6 membrane exhibited the highest water flux of 554.8 L m^−2^ h^−1^ during phase 1, which gradually declined to 528.1 L m^−2^ h^−1^ during phase VII. A similar pattern was observed for the M3 membrane, with a much lower initial water flux of 365.8 L m^−2^ h^−1^ during phase I and a final flux of 324.7 L m^−2^ h^−1^ during phase VII. These findings are in compliance with the higher porosity and pore size values for the M6 membrane as compared to the M3 sample. Bare PES membrane (M0) exhibited the lowest fluxes during these test experiments: the water flux declined from 162.3 L m^−2^ h^−1^ during phase 1 to 125.3 L m^−2^ h^−1^ during phase VII.

Figure 9b presents the normalized flux data for selected PES hybrid UF membranes. As seen, the M6 membrane exhibited the highest flux recovery compared to M0 and M3 membranes. This behavior might be attributed to the lower fouling of the M6 sample during HA filtration due to improved hydrophilicity of the PES/GO membrane, which minimized the adsorption of HA macromolecules on the membrane surface and within the porous membrane matrix.

#### 3.3.3. HA Rejection

**Figure 10:**
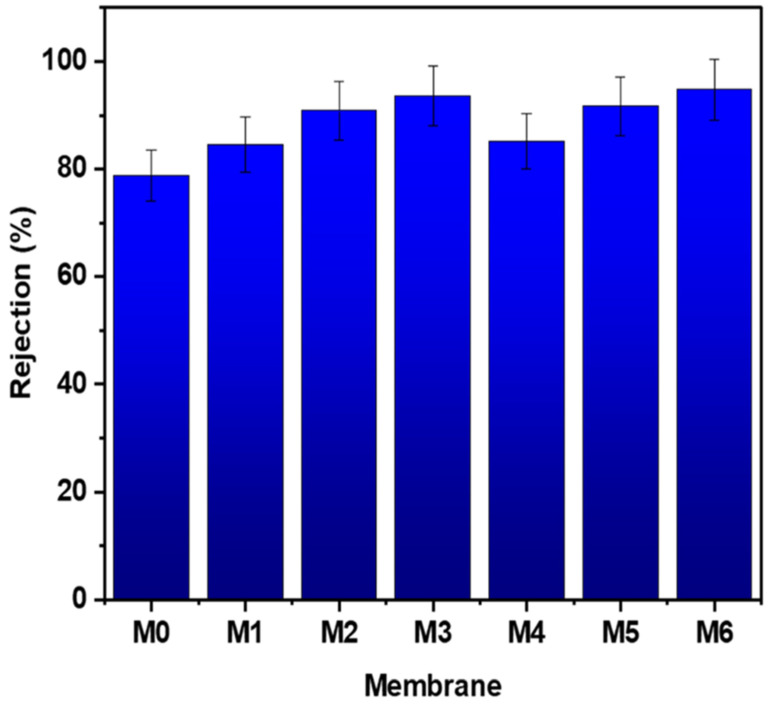
HA rejection with different PES hybrid membranes at pH = 7.

Figure 10 presents the HA rejection for different PES hybrid membranes. It was revealed that the PES/MWCNTs and PES/GO membranes exhibited higher HA rejection, up to 95%, compared to the bare PES membrane. This indicates that the removal efficiency of HA through the PES hybrid membranes was significantly improved by the addition of carbonaceous nanoparticles. The HA rejection percentage using M0, M3, and M6 membranes was 78.8%, 93.6%, and 94.5%, respectively. It might be assumed that a hydration layer is formed on the surface and in the pores of more hydrophilic PES/MWCNTs and PES/GO membranes, which thereby enhances the membrane rejection [6]. The higher HA removal with PES hybrid membranes compared to the bare PES membrane might also account for the enhanced electrostatic repulsive forces between the oxygen-containing functional groups of MWCNTs or GO and HA molecules [40,41].

#### 3.3.4. Flux Recovery of PES Hybrid Membranes

**Figure 11:**
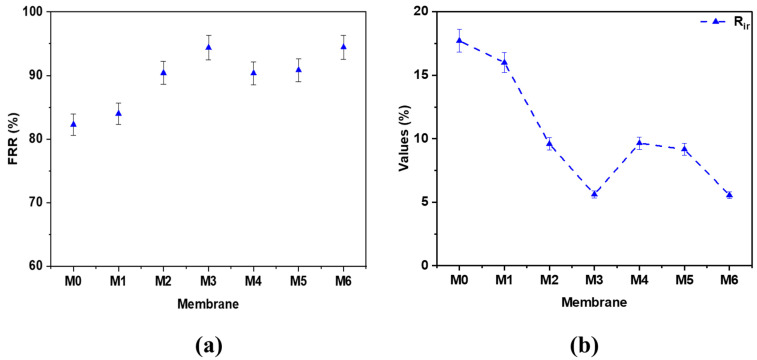
(**a**) Flux recovery ratios (FRR), and (**b**) irreversible resistance (R_ir_) for bare PES, PES/MWCNTs, and PES/GO hybrid membranes after ultrafiltration of HA solution (50 mg L^−1^, pH = 7) and subsequent washing with DW.

Figure 11a shows the flux recovery ratios of the prepared PES hybrid membranes. Among all the prepared membranes, the bare PES membrane (M0) demonstrated the smallest FRR value of 82.3%. This could be explained by the hydrophobic interaction between the HA molecules and the PES membrane matrix, resulting in membrane fouling. The FRR value was observed to increase with the incremental addition of MWCNTs and GO content to the PES matrix. In the case of PES/MWNCTs hybrid membranes, the flux recovery rates were seen to increase up to 90.4% by enhancing the concentration of MWCNTs to 1 wt.%. The M6 membrane with 1 wt.% GO exhibited the highest FRR values of 94.4%. The high FRR values of M3 and M6 membranes mainly accounted for the improved antifouling ability of the PES membrane by MWCNTs and GO addition due to higher hydrophilicity of PES hybrid membranes, which in turn reduced the hydrophobic interaction between the HA molecules and the PES matrix, resulting in reduced membrane fouling.

Figure 11b shows the irreversible resistance (R_ir_) of the prepared PES hybrid membranes. As seen, the R_ir_ for the bare PES membrane is comparably higher than the PES hybrid membranes modified with either MWCNTs and GO nanoadditives. It was also noticed that the R_ir_ decreases with increasing loading of MWCNTs and GO. It is worth mentioning that these behaviors are accounted for the weak binding of HA over the surface of the membrane and/or within the pores of the membrane, which could be easily removed by facile cleaning procedures using water flow. GO-incorporated PES membranes revealed lesser fouling capacities due to the hydrophobic adsorption between the HA and more hydrophilic membrane surface.

#### 3.3.5. Comparative Study

Table 3 shows a summary of the reported literature on PES UF membranes modified with MWCNTs or GO for the removal of different foulants such as BSA, HA, methyl red, reactive red 195, reactive blue 19, and oils. The table clearly suggests that the membrane performance is highly dependent on the physiochemical properties of the fabricated membranes. Regardless of the filler type and concentration of nano-fillers, the UF membranes with higher porosity and pore size and lower WCA, demonstrated better pure water flux and pollutants rejection. These outcomes are mainly attributed to the improved hydrophilicity and permeate channels by the use of MWCNTs or GO nanoparticles. Although the incorporation of both carbonaceous material into the PES matrix have demonstrated excellent physicochemical properties and membrane performance in previous studies, the results do not show a clear indication of which nanofiller material is better. According to our study, the GO blend PES membranes were more effective compared with PES/MWNCTs hybrid membranes.

The FRR values of both PES/MWNCTs and PES/GO membranes obtained in the current study were seen to be in compliance with those in the literature. It is also worth noting that even with the functionalization or the modification of MWCNTs and GO materials carried out in previous studies, we managed to exhibit highly porous and hydrophilic hybrid UF membranes with the addition of PVP, which served as a pore-forming agent with MWCNTs and GO nanoadditives.

## 4. Conclusions

In this study, the PES membranes incorporated with different loadings of MWCNTs and GO nanoadditives ranging from 0.1 wt.% to 1 wt.% have been prepared, characterized, and tested for HA removal from water. It was found that PES/hybrid membranes showed better permeability and higher HA rejection compared to the bare PES membrane. This is mainly accounted for the higher porosity, pore size, water uptake, and lower WCA of the modified membranes. The M6 membrane comprising 1 wt.% GO nanosheets achieved a DW and HA flux of 554.8 and 217.0 L m^−2^ h^−1^, 3.45- and 2-folds higher than the bare membrane, respectively. In addition, the HA rejection of M3 and M6 membranes was found to be 90.8% and 94.8%, respectively. It should be noted that M3 and M6 membranes demonstrated excellent antifouling membrane abilities, with FRR reaching ~90.8% and 94.4%, respectively. To conclude, although the addition of both MWCNTs and GO nanoadditives to the PES matrix exhibited tremendous results in terms of physiochemical properties and membrane performance, the GO-incorporated PES UF membranes was found to demonstrate better performance than MWCNTs modified membranes under same fabrication protocol and experimental testing conditions. This was mainly accredited the abundance of oxygen related functional groups onto the surface of GO particles and the impedance of GO within the PES membrane structure leading to better performance than the MWCNTs. 

## Figures and Tables

**Table 1:** Composition of casting solutions of PES UF membranes.

Membrane	PES (wt.%)	PVP (wt.%)	Nanoadditives	Nanoadditive Loading (wt.%)
M0	16	2	N/A	N/A
M1	16	2	MWCNTs	0.1
M2	16	2	MWCNTs	0.5
M3	16	2	MWCNTs	1.0
M4	16	2	GO	0.1
M5	16	2	GO	0.5
M6	16	2	GO	1.0

**Table 2:** Physicochemical properties for the hybrid ultrafiltration membranes: water uptake; φ, contact angles; θ and free surface energy; $-\Delta G_{LS}$.

Membrane	φ (%)	θ (°)	$-\Delta G_{LS}$
M0	74.62	72.3	94.9
M1	78.76	64.8	103.8
M2	79.19	63.8	104.9
M3	79.73	61.1	107.9
M4	79.72	63.4	105.4
M5	80.08	62.8	106.1
M6	81.65	60.3	108.9

**Table 3:** Comparison between different PES-based UF hybrid membranes incorporated with MWCNTs and GO nanoadditives with those prepared in this study for water treatment applications.

Membrane (wt.%)	Membrane Preparation Method	Physicochemical Properties			Water Flux (LMH) and Pollutant Rejection (%)	FRR (%)	Ref.
		Porosity (%)	Mean Pore Size (nm)	WCA (°)			
PES/GO (0.008)	Phase inversion assisted by a direct current electric field	80	23.2	50.7	289.86 at 4 bar 86.58% Methyl red	89.18	[42]
PES/GA/GO (5)	Phase inversion	~85	66.4	~58–59	~200 at 1 bar ~80–85% BSA	75	[43]
GO/Fe3O4/PES (2)	Phase inversion	~75	~30	66.1	175.6 at 1 bar 98.0% BSA	87.9	[44]
M-GO/Fe3O4/PES (2)	Phase inversion	~86	~32	53.9	252.7 at 1 bar 92.3% BSA	80.1	
CGO/PES (0.5)	Non-solvent-induced phase separation	N/A	7.2	53.2	82.6 at 2 bar 99.8% BSA	92.1	[45]
PES-GO (0.6)	Phase inversion	61.6	N/A	46.9	161 at 1 bar 97% HA 99.5% BSA	85	[46]
PES/GO (1)	Non-solvent-induced phase separation	76	68.5	60.3	554.8 at 1 bar 94.8% HA	94.4	This work
MoS2/O-MWCNTs/PES (0.75)	Phase inversion	81.7	25.1	49.6	192.2 at 3 bar 98.4% Reactive red 195 93.5% Reactive blue 19 99.0% BSA	60.8	[47]
PES/ZnO-MWCNTs (0.5)	Non-solvent-induced phase separation	46.02	58.81	58.7	57.1 at 1 bar 88.5% HA	77.18	[48]
PES/SPSf/O-MWCNT (0.05)	Non-solvent-induced phase separation	88	40.9	~50	553 at 1 bar 100% BSA	~93	[49]
SiO2-f-MWCNTs/PES (2)	Phase inversion	~70	~65	55.3	293 at 0.7 bar 85.6% HA 97% oil	99	[50]
f-CNT/PES (2)	Phase inversion	74	~86–87	57	~600 at 1 bar >95% BSA	>90	[51]
F-MWCNTs/PES (1)	Phase inversion	N/A	N/A	51.9	~185 at 3 bar ~86–87% BSA	46	[39]
PES/GA/OMWCNTs (3)	Phase inversion	~82	~68	~64	~520 at 1 bar ~80% BSA	N/A	[15]
PES/MWCNTs (0.5)	Non-solvent-induced phase separation	61.5	61.5	63.8	450 at 1 bar 90.8% HA	90.4	This work

## Data Availability

Not applicable.

## References

[1] Almanassra I.W., Kochkodan V., Mckay G., Atieh M.A., Al-Ansari T. (2021). Kinetic and Thermodynamic Investigations of Surfactants Adsorption from Water by Carbide-Derived Carbon. J. Environ. Sci. Heal. Part A Toxic/Hazard. Subst. Environ. Eng..

[2] Cevallos-Mendoza J., Amorim C.G., Rodríguez-Díaz J.M., Montenegro M.D.C.B.S.M. (2022). Removal of Contaminants from Water by Membrane Filtration: A Review. Membranes.

[3] Yu T., Zhou J., Liu F., Xu B.M., Pan Y. (2022). Recent Progress of Adsorptive Ultrafiltration Membranes in Water Treatment—A Mini Review. Membranes.

[4] Sabri S., Najjar A., Manawi Y., Eltai N.O., Al-Thani A., Atieh M.A., Kochkodan V. (2019). Antibacterial Properties of Polysulfone Membranes Blended with Arabic Gum. Membranes.

[5] Siagian U.W.R., Khoiruddin K., Wardani A.K., Aryanti P.T.P., Widiasa I.N., Qiu G., Ting Y.P., Wenten I.G. (2021). High-Performance Ultrafiltration Membrane: Recent Progress and Its Application for Wastewater Treatment. Curr. Pollut. Rep..

[6] Kallem P., Othman I., Ouda M., Hasan S.W., AlNashef I., Banat F. (2021). Polyethersulfone Hybrid Ultrafiltration Membranes Fabricated with Polydopamine Modified ZnFe2O4 Nanocomposites: Applications in Humic Acid Removal and Oil/Water Emulsion Separation. Process Saf. Environ. Prot..

[7] Ahmad A.L., Abdulkarim A.A., Ooi B.S., Ismail S. (2013). Recent Development in Additives Modifications of Polyethersulfone Membrane for Flux Enhancement. Chem. Eng. J..

[8] Manawi Y., Kochkodan V., Mahmoudi E., Johnson D.J., Mohammad A.W., Atieh M.A. (2017). Characterization and Separation Performance of a Novel Polyethersulfone Membrane Blended with Acacia Gum. Sci. Rep..

[9] Alkhouzaam A., Qiblawey H. (2021). Novel Polysulfone Ultrafiltration Membranes Incorporating Polydopamine Functionalized Graphene Oxide with Enhanced Flux and Fouling Resistance. J. Membr. Sci..

[10] Algamdi M.S., Alsohaimi I.H., Lawler J., Ali H.M., Aldawsari A.M., Hassan H.M.A. (2019). Fabrication of Graphene Oxide Incorporated Polyethersulfone Hybrid Ultrafiltration Membranes for Humic Acid Removal. Sep. Purif. Technol..

[11] Rambabu K., Bharath G., Monash P., Velu S., Banat F., Naushad M., Arthanareeswaran G., Loke Show P. (2019). Effective Treatment of Dye Polluted Wastewater Using Nanoporous CaCl2 Modified Polyethersulfone Membrane. Process Saf. Environ. Prot..

[12] Low Z.X., Wang Z., Leong S., Razmjou A., Dumée L.F., Zhang X., Wang H. (2015). Enhancement of the Antifouling Properties and Filtration Performance of Poly(Ethersulfone) Ultrafiltration Membranes by Incorporation of Nanoporous Titania Nanoparticles. Ind. Eng. Chem. Res..

[13] Kallem P., Bharath G., Rambabu K., Srinivasakannan C., Banat F. (2021). Improved Permeability and Antifouling Performance of Polyethersulfone Ultrafiltration Membranes Tailored by Hydroxyapatite/Boron Nitride Nanocomposites. Chemosphere.

[14] Kallem P., Ouda M., Bharath G., Hasan S.W., Banat F. (2022). Enhanced Water Permeability and Fouling Resistance Properties of Ultrafiltration Membranes Incorporated with Hydroxyapatite Decorated Orange-Peel-Derived Activated Carbon Nanocomposites. Chemosphere.

[15] Najjar A., Sabri S., Al-Gaashani R., Atieh M.A., Kochkodan V. (2019). Antibiofouling Performance by Polyethersulfone Membranes Cast with Oxidized Multiwalled Carbon Nanotubes and Arabic Gum. Membranes..

[16] Ouda M., Hai A., Krishnamoorthy R., Govindan B., Othman I., Kui C.C., Choi M.Y., Hasan S.W., Banat F. (2022). Surface Tuned Polyethersulfone Membrane Using an Iron Oxide Functionalized Halloysite Nanocomposite for Enhanced Humic Acid Removal. Environ. Res..

[17] Lewis J., Al-sayaghi M.A.Q., Buelke C., Alshami A. (2021). Activated Carbon in Mixed-Matrix Membranes. Sep. Purif. Rev..

[18] Van Der Bruggen B. (2009). Chemical Modification of Polyethersulfone Nanofiltration Membranes: A Review. J. Appl. Polym. Sci..

[19] Jiang Y., Zeng Q., Biswas P., Fortner J.D. (2019). Graphene Oxides as Nanofillers in Polysulfone Ultrafiltration Membranes: Shape Matters. J. Membr. Sci..

[20] Seetharaman S., Raghu S.C., Velan M., Ramya K., Mahabadi K.A. (2015). Comparison of the Performance of Reduced Graphene Oxide and Multiwalled Carbon Nanotubes Based Sulfonated Polysulfone Membranes for Electrolysis Application. Polym. Compos..

[21] Zhang J., Xu Z., Shan M., Zhou B., Li Y., Li B., Niu J., Qian X. (2013). Synergetic Effects of Oxidized Carbon Nanotubes and Graphene Oxide on Fouling Control and Anti-Fouling Mechanism of Polyvinylidene Fluoride Ultrafiltration Membranes. J. Membr. Sci..

[22] Kadhim R.J., Al-Ani F.H., Al-Shaeli M., Alsalhy Q.F., Figoli A. (2020). Removal of Dyes Using Graphene Oxide (Go) Mixed Matrix Membranes. Membranes.

[23] Zhang J., Xu Z., Mai W., Min C., Zhou B., Shan M., Li Y., Yang C., Wang Z., Qian X. (2013). Improved Hydrophilicity, Permeability, Antifouling and Mechanical Performance of PVDF Composite Ultrafiltration Membranes Tailored by Oxidized Low-Dimensional Carbon Nanomaterials. J. Mater. Chem. A.

[24] Shevate R., Kumar M., Karunakaran M., Hedhili M.N., Peinemann K.V. (2017). Polydopamine/Cysteine Surface Modified Isoporous Membranes with Self-Cleaning Properties. J. Membr. Sci..

[25] Abdi J., Vossoughi M., Mahmoodi N.M., Alemzadeh I. (2017). Synthesis of Metal-Organic Framework Hybrid Nanocomposites Based on GO and CNT with High Adsorption Capacity for Dye Removal. Chem. Eng. J..

[26] Sun L., Wang M., Li W., Luo S., Wu Y., Ma C., Liu S. (2020). Carbon Material–Immobilized Ionic Liquids Were Applied on Absorption of Hg^2+^ from Water Phase. Environ. Sci. Pollut. Res..

[27] Parvez K., Wu Z.S., Li R., Liu X., Graf R., Feng X., Müllen K. (2014). Exfoliation of Graphite into Graphene in Aqueous Solutions of Inorganic Salts. J. Am. Chem. Soc..

[28] Moosa A.A., Abed M.S. (2021). Graphene Preparation and Graphite Exfoliation. Turk. J. Chem..

[29] Kumar V., Kumar A., Lee D.J., Park S.S. (2021). Estimation of Number of Graphene Layers Using Different Methods: A Focused Review. Materials.

[30] Nguyen V.T., Le H.D., Nguyen V.C., Ngo T.T.T., Le D.Q., Nguyen X.N., Phan N.M. (2013). Synthesis of Multi-Layer Graphene Films on Copper Tape by Atmospheric Pressure Chemical Vapor Deposition Method. Adv. Nat. Sci. Nanosci. Nanotechnol..

[31] Hwangbo Y., Lee C.K., Mag-Isa A.E., Jang J.W., Lee H.J., Lee S.B., Kim S.S., Kim J.H. (2014). Interlayer Non-Coupled Optical Properties for Determining the Number of Layers in Arbitrarily Stacked Multilayer Graphenes. Carbon.

[32] Krishnamoorthy R., Sagadevan V. (2015). Polyethylene Glycol and Iron Oxide Nanoparticles Blended Polyethersulfone Ultrafiltration Membrane for Enhanced Performance in Dye Removal Studies. E-Polymers.

[33] Behdarvand F., Valamohammadi E., Tofighy M.A., Mohammadi T. (2021). Polyvinyl Alcohol/Polyethersulfone Thin-Film Nanocomposite Membranes with Carbon Nanomaterials Incorporated in Substrate for Water Treatment. J. Environ. Chem. Eng..

[34] Zinadini S., Zinatizadeh A.A., Rahimi M., Vatanpour V., Zangeneh H. (2014). Preparation of a Novel Antifouling Mixed Matrix PES Membrane by Embedding Graphene Oxide Nanoplates. J. Membr. Sci..

[35] Zhu K., Wang G. (2018). Fabrication of High-Performance Ultrafiltration Membranes Using Zwitterionic Carbon Nanotubes and Polyethersulfone. High Perform. Polym..

[36] Mehrparvar A., Rahimpour A., Jahanshahi M. (2014). Modified Ultrafiltration Membranes for Humic Acid Removal. J. Taiwan Inst. Chem. Eng..

[37] Celik E., Park H., Choi H., Choi H. (2011). Carbon Nanotube Blended Polyethersulfone Membranes for Fouling Control in Water Treatment. Water Res..

[38] Han M.J., Nam S.T. (2002). Thermodynamic and Rheological Variation in Polysulfone Solution by PVP and Its Effect in the Preparation of Phase Inversion Membrane. J. Membr. Sci..

[39] Rahimpour A., Jahanshahi M., Khalili S., Mollahosseini A., Zirepour A., Rajaeian B. (2012). Novel Functionalized Carbon Nanotubes for Improving the Surface Properties and Performance of Polyethersulfone (PES) Membrane. Desalination.

[40] Ye W., Liu H., Lin F., Lin J., Zhao S., Yang S., Hou J., Zhou S., Van Der Bruggen B. (2019). High-Flux Nanofiltration Membranes Tailored by Bio-Inspired Co-Deposition of Hydrophilic g-C3N4 Nanosheets for Enhanced Selectivity towards Organics and Salts. Environ. Sci. Nano.

[41] Li X., Li J., Fang X., Bakzhan K., Wang L., Van der Bruggen B. (2016). A Synergetic Analysis Method for Antifouling Behavior Investigation on PES Ultrafiltration Membrane with Self-Assembled TiO2 Nanoparticles. J. Colloid Interface Sci..

[42] Wang X., Feng M., Liu Y., Deng H., Lu J. (2019). Fabrication of Graphene Oxide Blended Polyethersulfone Membranes via Phase Inversion Assisted by Electric Field for Improved Separation and Antifouling Performance. J. Membr. Sci..

[43] Najjar A., Sabri S., Al-Gaashani R., Kochkodan V., Atieh M.A. (2019). Enhanced Fouling Resistance and Antibacterial Properties of Novel Graphene Oxide-Arabic Gum Polyethersulfone Membranes. Appl. Sci..

[44] Mirzaei M., Mohammadi T., Kasiri N., Tofighy M.A. (2021). Fabrication of Magnetic Field Induced Mixed Matrix Membranes Containing GO/Fe_3_O_4_ nanohybrids with Enhanced Antifouling Properties for Wastewater Treatment Applications. J. Environ. Chem. Eng..

[45] Kong S., Lim M., Shin H., Baik J.H., Lee J.C. (2020). High-Flux and Antifouling Polyethersulfone Nanocomposite Membranes Incorporated with Zwitterion-Functionalized Graphene Oxide for Ultrafiltration Applications. J. Ind. Eng. Chem..

[46] Lemos H.G., Ragio R.A., Conceição A.C.S., Venancio E.C., Mierzwa J.C., Subtil E.L. (2021). Assessment of Mixed Matrix Membranes (MMMs) Incorporated with Graphene Oxide (GO) for Co-Treatment of Wastewater and Landfill Leachate (LFL) in a Membrane Bioreactor (MBR). Chem. Eng. J..

[47] Arefi-Oskoui S., Khataee A., Jabbarvand Behrouz S., Vatanpour V., Haddadi Gharamaleki S., Orooji Y., Safarpour M. (2022). Development of MoS2/O-MWCNTs/PES Blended Membrane for Efficient Removal of Dyes, Antibiotic, and Protein. Sep. Purif. Technol..

[48] Pang W.Y., Ahmad A.L., Zaulkiflee N.D. (2019). Antifouling and Antibacterial Evaluation of ZnO/MWCNT Dual Nanofiller Polyethersulfone Mixed Matrix Membrane. J. Environ. Manag..

[49] Gumbi N.N., Hu M., Mamba B.B., Li J., Nxumalo E.N. (2018). Macrovoid-Free PES/SPSf/O-MWCNT Ultrafiltration Membranes with Improved Mechanical Strength, Antifouling and Antibacterial Properties. J. Membr. Sci..

[50] Hegab H.M., Elaraby A., Ibrahim Y., Elmekawy A., Marzooqi F., Aljundi I.H., Hasan S.W. (2022). Designing of Amino Silica Covalently Functionalized Carboxylic Multi-Wall Carbon Nanotubes-Based Polyethersulfone Membranes for Enhancing Oily Wastewater Treatment. J. Environ. Chem. Eng..

[51] Wang W., Zhu L., Shan B., Xie C., Liu C., Cui F., Li G. (2018). Preparation and Characterization of SLS-CNT/PES Ultrafiltration Membrane with Antifouling and Antibacterial Properties. J. Membr. Sci..

